# The Antifibrotic and the Anticarcinogenic Activity of Capsaicin in Hot Chili Pepper in Relation to Oral Submucous Fibrosis

**DOI:** 10.3389/fphar.2022.888280

**Published:** 2022-05-04

**Authors:** Zoufang Huang, Mohit Sharma, Aparna Dave, Yuqi Yang, Zhe-Sheng Chen, Raghu Radhakrishnan

**Affiliations:** ^1^ Ganzhou Key Laboratory of Hematology, Department of Hematology, the First Affiliated Hospital of Gannan Medical University, Ganzhou, China; ^2^ Department of Oral Pathology, SGT Dental College Hospital and Research Institute, Gurugram, India; ^3^ College of Pharmacy and Health Sciences, St. John’s University, New York, NY, United States; ^4^ Department of Oral Pathology, Manipal College of Dental Sciences, Manipal Academy of Higher Education, Manipal, India

**Keywords:** capsaicin, burning pain, chili, oral submucous fibrosis, antifibrotic, anticarcinogen

## Abstract

A burning sensation on eating spicy foods purportedly supports the role of capsaicin, an active component of chili peppers, in the etiology of oral submucous fibrosis (OSF). Although the mast cell mediators and activated P2X receptors induce a constant burning sensation through an ATP-dependent mechanism, it is the activation of the transient receptor potential vanilloid 1 (TRPV-1) receptor by capsaicin that aggravates it. The molecular basis for the burning pain in OSF is thus attributable to the activation of TRPV1. There is overwhelming evidence that confirms capsaicin has more of a protective role in attenuating fibrosis and is potentially therapeutic in reversing conditions linked to collagen accumulation. The activation of TRPV-1 by capsaicin increases intracellular calcium ([Ca^2+^]_i_), upregulates AMP-activated protein kinase (AMPK) and Sirtuin-1 (SIRT-1), to enrich endothelium-dependent vasodilation via endothelial nitric oxide synthase (eNOS). The induction of vasodilation induces antifibrotic effects by alleviating hypoxia. The antifibrotic effects of capsaicin are mediated through the upregulation of antioxidant enzymes, downregulation of inflammatory genes and suppression of new collagen fibril formation. Capsaicin also demonstrates an anticarcinogenic effect by upregulating the cytotoxic T cells and downregulating regulatory T cells through the inhibition of angiogenesis and promotion of apoptosis. Judicious administration of capsaicin with an appropriate delivery mechanism may have therapeutic benefits in reducing pain sensation, rendering antifibrotic effects, and preventing the malignant transformation of OSF. This paper provides an overview of the molecular basis of capsaicin and its therapeutic application as an antifibrotic and anticarcinogenic agent for the treatment of OSF.

## Graphical Abstract

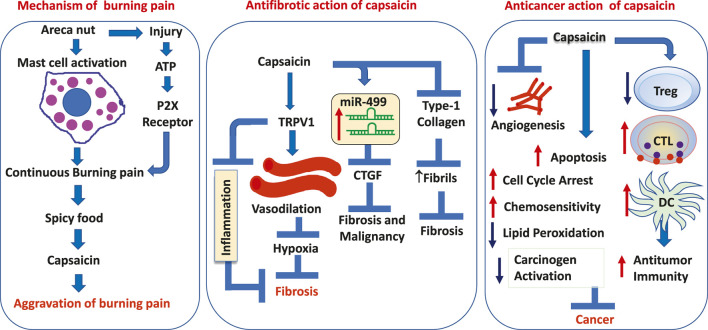

## Introduction

One of the telltale symptoms of oral submucous fibrosis (OSF) is a burning sensation in the mouth when eating spicy foods. Vesicular stomatitis/blister formation following the intake of foods laced with chilies (Capsicum annum and Capsicum frutescence) has led to the hypothesis that supports the etiological role of chilies in the development of OSF. This was reinforced by Sirsat and Khanolkar in 1960 (Sirsat and Khanolkar, 1960a; Sirsat and Khanolkar, 1960b), who were able to produce a mucosal connective tissue response similar to OSF by topical application of capsaicin (8-methyl-N-vanillyl-6-nonenamide), the active ingredient in chilies, in protein depleted and vitamin B deficient Wistar rats (Sirsat and Khanolkar, 1960a). Histologically, the epithelial changes and sub-epithelial hyalinization were reminiscent of OSF, but no changes were noted in the deeper connective tissue characteristic of fibrosis (Sirsat and Khanolkar, 1960a; Sirsat and Khanolkar, 1960b). Since these alterations were non-specific and indicative of local reaction to irritants, the authors were guarded in proposing chilies as the causative factor in OSF (Sirsat and Khanolkar, 1960a; Murti et al., 1995). Thus, they reported that ‘conditioned mucosa’ owing to vitamin and protein deficiency might make the mucosa susceptible to OSF. However, it has been observed that the dietary state may not be the primary factor as patients with advanced OSF have difficulty having a normal diet. Also, there are no interventional studies that support the protective effects of nutritional supplements against OSF (Cox and Walker, 1996).

Several investigators have supported the chili hypothesis (Sirsat and Khanolkar, 1960a; Sirsat and Pindborg, 1967; Shiau and Kwan, 1979) with some suggesting an allergic reaction to chilies (Sirsat and Khanolkar, 1960a; Pindborg et al., 1964; Pindborg and Singh, 1964; Pindborg and Sirsat, 1966; Sirsat and Pindborg, 1967; Pindborg et al., 1968; Shiau and Kwan, 1979). Hypersensitivity to chilies and a susceptible mucosa result in increased eosinophilia in tissue and serum suggesting that OSF arises as a result of an allergic reaction to chilies (Desa, 1957; Sirsat and Khanolkar, 1960a; Rao, 1962; Pindborg et al., 1964; Pindborg and Singh, 1964; Pindborg and Sirsat, 1966; Wahi et al., 1966; Sirsat and Pindborg, 1967; Pindborg et al., 1968; Hamner et al., 1971; Shiau and Kwan, 1979). In this paper, we provide an explanation for the continuous burning pain in OSF and attempt to correct the myth that chili is an etiologic factor in the development of OSF. We explain the molecular mechanisms of capsaicin-mediated antifibrotic and anticarcinogenic effects and draw attention to the therapeutic use of capsaicin in reversing fibrosis and preventing the malignant transformation of OSF.

## Alternative Chili Hypothesis in the Development of OSF

The role of chilies in the development of OSF has been widely discussed in the literature. While the formation of vesicles and secondary eosinophilia has been considered to be the manifestation of an allergic response to chili (Pindborg and Singh, 1964; Pindborg and Sirsat, 1966), several investigators have refuted this association (Desa, 1957; Rao, 1962; Wahi et al., 1966; Hamner et al., 1971; Shiau and Kwan, 1979; Gupta et al., 1980). Even though the formation of vesicles occurred within 3 hours of consumption of food strongly laced with chili (Pindborg and Sirsat, 1966), only 30% of OSF patients showed above normal absolute eosinophil counts (Wahi et al., 1966).

In a case series analysis by Desa (1957) only 6/64 patients with OSF (9.4%) reported excessive use of chilies (Desa, 1957). Contesting the chili hypothesis, Wahi et al. (1966) showed that neither the amount nor the duration of chili consumption contributed to OSF progression (Wahi et al., 1966). Likewise, Hamner et al. (1971) failed to produce OSF in hamster cheek cells with topical application of 2% capsaicin dissolved in mineral oil or freshly ground chili powder 3 times per week for 2-years (Hamner et al., 1971). Furthermore, OSF is not reported in Mexico and South America, where the dietary intake of chilies equals or even exceeds that in India (Cox and Walker, 1996). Shiau and Kwan (1979) in their report on 35 OSF patients in Taiwan did not see any causal association between chili consumption and OSF (Shiau and Kwan, 1979). Gupta et al. (1980) in their 10-years follow-up of Indian villagers could not correlate the disease process to any other dietary factors except the use of areca nut (AN) (Gupta et al., 1980). Seedat and van Wyk (1988) could not find any association between OSF and the use of tobacco, lime, and chili among those who chewed AN (Seedat and van Wyk, 1988).

### Continuous Burning Pain in OSF: Molecular Mechanisms

Patients with OSF suffer constant burning pain which is often aggravated by the intake of spicy food (Desa, 1957; Murti et al., 1995; Kumar et al., 2020). The burning pain is present in every stage of the disease and is significantly worse in clinical stage III (Kumar et al., 2020). Several attempts have been made to address the plausible mechanism and active agents that cause the burning mucosa in OSF (Lu and Insel, 2014; Feller et al., 2017; Yu et al., 2018; Mendivil et al., 2019). The burning pain that the patients experience in OSF, particularly when the buccal and labial mucosa is involved is alleviated with the use of local anesthetic rinses. It was thus suggested that the burning pain in OSF was due to the irritation of nerve endings and inflammation of the nerve secondary to changes in the connective tissue (Desa, 1957; Kumar et al., 2020). Such an altered mucosa showed a decrease in the number of small-diameter nerve fibers and the activation of transient receptor potential vanilloid 1 (TRPV1) receptors, P2X3 receptors, and nerve growth factor (NGF) (Feller et al., 2017).

**FIGURE 1:**
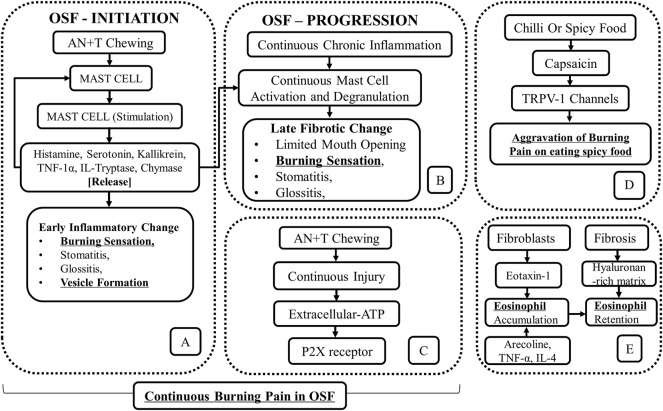
Schematic outline of the role of **(A,B)** mast cells in continuous burning pain and vesicles formation in OSF (Adapted and Modified from Pujari and Vidya, 2013), **(C)** P2X receptors in a continuous burning pain in OSF, **(D)** chili or spicy food in aggravation of burning pain in OSF. **(E)** The accumulation of eosinophils in OSF as a consequence of the process of fibrosis and as a result of irritation by areca nut byproducts.

Another explanation for the persistent burning pain in OSF relates to mast cell activation by areca nut and tobacco chewing (Pujari and Vidya, 2013; Kumar et al., 2020) (Figures 1A, B). With increased mast cell density (MCD) noted with the progression of the disease, it was proposed that areca nut by-products cause the release of mast cell mediators like histamine, serotonin, kallikrein, TNF-1α, IL-1, tryptase, and chymase. The release of mast cell mediators was reported to produce a burning sensation secondary to stomatitis, glossitis, and vesicle formation (Pujari and Vidya, 2013; Kumar et al., 2020) (Figure 1A). Further mast cell activity and degranulation resulted in fibrotic change, characterized by limited mouth opening (Pujari and Vidya, 2013) (Figure 1B).

The molecular mechanism for the burning sensation in OSF has been attributed to the activation of the P2X receptor. While it was noted that fibrosis is mediated by P2X receptors present on fibroblasts in an autocrine manner (Lu and Insel, 2014), their presence on nerve fibers (Feller et al., 2017), evoked a continuous burning pain due to the activation of the P2X receptor by extracellular ATP (Lu and Insel, 2014; Feller et al., 2017). The latter is released through connexin (Cxs)/pannexin (Panx) hemichannels in areas of chronic tissue injury (Figure 1C) (Lu and Insel, 2014).

### TRPV-1 Activation and the Aggravation of Burning Pain

Heat, capsaicin, and H^+^ ions present in acidic food are the known agonists of TRPV1 receptors, which mediate the sensation of burning pain (Yu et al., 2018). The best-known TRPV-1 activator among these stimulants is capsaicin, present in chilies and peppers (McCarty et al., 2015; Mendivil et al., 2019). The aggravation of burning pain in OSF by hot, spicy, and acidic foods has been ascribed to TRPV1 stimulation (Yu et al., 2018; Mendivil et al., 2019). It has been established that the TRPV-1 receptor activation by capsaicin may result in the aggravation of burning pain than capsaicin/spicy food being a precursor for the disease (Figure 1D)

### Targetting the Pathways of Burning Pain in OSF

The burning pain in OSF can be alleviated by targetting the NGF pathway (Watson et al., 2008) and the P2X receptor pathway (Bernier et al., 2018). The drugs specifically targetting the P2X3 pathway like A317491, AF-219, IP5, and RO-85 can be envisaged as novel pharmacological agents for treating burning pain in OSF (Bernier et al., 2018). Several mast cell released factors like serotonin, kallikrein, histamine, tryptase, Substance-p, prostaglandins, TNF-α, IL-1 are the mediators of inflammatory pain (Sharma, 2005; Roman et al., 2014; Kempuraj et al., 2019; Paredes et al., 2019; Mailhot et al., 2020; Alexander et al., 2021; Salvo et al., 2021). The use of mast cell stabilizers like ketotifen (Meloto et al., 2021) can reduce the burning pain in OSF, by countering these mediators, besides having a beneficial effect on afflicted mucosa. The use of AZD5213 the inverse agonist of histamine three receptor and INCB38579 as an antagonist of histamine three receptor are the other therapeutic modalities for the control of pain in OSF (Shin et al., 2012; Alexander et al., 2021). The repeated application of capsaicin itself can lead to depletion of substance-p and receptor desensitization (Friedman et al., 2018).

### Eosinophilia and its Significance in OSF

The accumulation of eosinophils in OSF tissues has been attributed to the presence of a hyaluronan-rich matrix, which tends to retain eosinophils in the areas of fibrosis (Wight and Potter-Perigo, 2011). The eosinophil accumulation in OSF is attributable to the release of eotaxin-1, a potent eosinophil chemoattractant, from the gingival fibroblasts due to areca nut chewing along with the release of TNF-α and IL-4. However, the fact that eosinophilia is present in only 30% of OSF cases does not support the role of chili as an allergen causing OSF. Instead, increased eosinophilia in OSF is a result of the fibrotic process rather than an allergic reaction. This explains the association between asthma and decreased lung function among betel chewers at an adjusted odds ratio of 2.05 with a 95% confidence interval (Wang TN et al., 2014) (Figure 1E).

## The Role of Capsaicin as an Antifibrotic Agent

**FIGURE 2:**
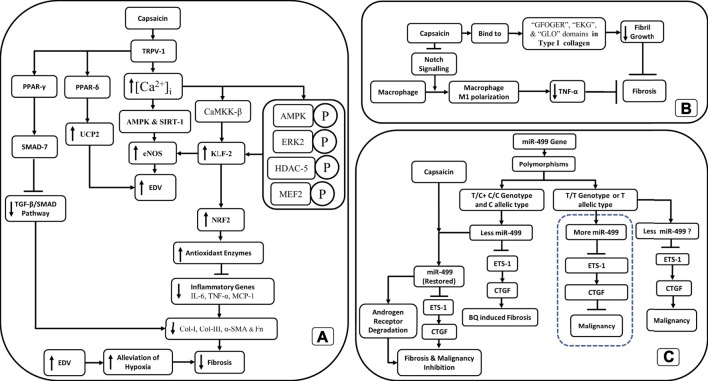
Schematic outline of the antifibrotic action of capsaicin through **(A)** TRPV-1 channels, **(B)** inhibition of fibril growth and decreased M1 macrophage polarization. **(C)** The restoration of miR-499 through capsaicin exposure inhibits both fibrosis and malignancy in OSF.

While the role of capsaicin in fibrosis has been challenged, several studies have shown that capsaicin has proven antifibrotic properties (Wang Q et al., 2014; Perumal et al., 2015; Choi et al., 2017; Mendivil et al., 2019; Sheng et al., 2020). Capsaicin activation of TRPV-1 increases intracellular calcium ([Ca^2+^]_i_), upregulates AMP-activated protein kinase (AMPK), and SIRT-1, which then upregulates endothelial nitric oxide synthase (eNOS) (McCarty et al., 2015), thus improving the endothelium-dependent vasodilation (EDV). Improved EDV could lead to alleviation of hypoxia and inhibition of fibrosis (Figure 2A).

Increased [Ca^2+^]_i_ also upregulates endothelial-specific transcription factor Krüppel-like Factor 2 (KLF2) through the activation of Ca^2+^/calmodulin-dependent protein kinase kinase-β (CaMKK-β) and downstream phosphorylation of AMP-activated protein kinase (AMPK), extracellular signal-regulated kinase 2 (ERK2), histone deacetylase-5 (HDAC-5), and myocyte enhancer factor-2 (MEF2) (McCarty et al., 2015). KLF2 also upregulates eNOS, antioxidant enzymes responsive to nuclear factor erythroid 2-related factor 2 (NRF2), and thrombomodulin. The upregulation of these antioxidant enzymes downregulates inflammatory genes like nuclear factor-kappa B (NF-κB), IL-6, TNF-α, and Monocyte Chemoattractant Protein-1 (MCP-1) (McCarty et al., 2015; Choi et al., 2017). TRPV-1 upregulates uncoupling protein 2 (UCP2) through peroxisome proliferator-activated receptor delta (PPAR-δ). Augmented UCP2 has proven to be important in promoting EDV in mice (McCarty et al., 2015). Besides that, peroxisome proliferator-activated receptor gamma (PPAR-γ) is upregulated (McCarty et al., 2015), which mediates its antifibrotic effects by inhibiting the TGF-β1/Smad pathway through SMAD-7 (Choi et al., 2017). Inhibition of the TGF-β1/Smad pathway decreases collagen I (Col-I), collagen III, *a*-SMA, and fibronectin (Fn) deposits in the tissue (Wang Q et al., 2014; Choi et al., 2017) (Figure 2A).

In the liver, capsaicin has been shown to attenuate fibrosis through the inhibition of notch signaling, inhibiting M1-polarization of macrophages, and reducing TNF-α secretion (Sheng et al., 2020). Capsaicin along with sulforaphane synergistically inhibited fibrosis of the liver by upregulating PPAR-γ and Nrf2. This, in turn, upregulated catalase and downregulated proinflammatory cytokines like TGF-β1, TNF-α, IL-6, and Type I collagen (Mendivil et al., 2019). Nrf2 interacts with the antioxidant response element of the catalase gene to enhance its production (Mendivil et al., 2019).

Similarly, in benzo(a)pyrene-induced lung cancer the levels of ECM components like collagen, elastin, uronic acid, and glycosaminoglycans such as hyaluronic acid, chondroitin sulfate, keratan sulfate, dermatan sulfate were elevated, but upon treatment with capsaicin, they were significantly reduced (Cho et al., 2017). TRPV-1 activation protects against renal and cardiac fibrosis through inhibition of the TGF-β/Smad2/3 signaling pathway (Wang Q. et al., 2014; Yu et al., 2018). In the peritoneal macrophages, the anti-inflammatory activity of capsaicin is facilitated by IkB-α degradation (Kim et al., 2003). In the retina, capsaicin renders a protective effect on ischemia-induced injury (Wang et al., 2017). On the contrary, conjunctival epithelial cells possessing TRPV1 channels seem to be profibrotic and proinflammatory (Yang et al., 2013; Khajavi et al., 2014). This is attributable to corneal epithelial cells containing the highest density of capsaicin-sensitive TRPV1 channels in the human body (Muller et al., 2003; Marfurt et al., 2010).

In oral mucosa TRPV1 channels functions as an environment sensing protein (Takahashi et al., 2020). However, the effect of capsaicin is dose-dependent with lower doses being antifibrotic, and higher doses being profibrotic (Yu et al., 2018). The antifibrotic mechanism of capsaicin and the pathways involved have been discussed in Table 1.

**TABLE 1:** Potential antifibrotic mechanisms of capsaicin.

Agent	Pathway/Mediators/Mechanism	Tissue	References
**Capsaicin + sulforaphane**	↑PPAR-γ, ↑Nrf2, ↑Catalase, ↓TGF-β1, ↓TNF-α, ↓IL-6, ↓Col-I	Liver Fibrosis	Mendivil et al. (2019)
**Capsaicin**	↑PPAR-γ, ↓TGF-β, ↓SMAD-2,3, ↑SMAD7, ↓NF-κB, ↓TNF-α, ↓α-SMA, ↓Col-I	Liver Fibrosis	Choi et al. (2017)
**Capsaicin**	↑TRPV-1,↓TGF-β, ↓SMAD-2,3,↓CTGF, ↓MMP-2,4,13, ↓Fibronectin,↑Col-I&III,↓AT-II-induced-fibroblast proliferation	Cardiac Fibrosis	Wang Q. et al. (2014)
**Capsaicin**	↓Collagen Fibril Formation	Rat Tail Tendon	Perumal et al. (2015)
**Capsaicin**	↓IL-6, ↓IL-12, ↓TNF-α, ↓Hes-1, ↓Notch-1, ↓M-1 Macrophage Polarization	Liver Fibrosis	Sheng et al. (2020)
**Capsaicin**	↓Collagen,↓CS, ↓DS, ↓Elastin, ↓HA,↓KS, ↓UA	Lung Cancer	Cho et al. (2017)
**Capsaicin**	↓ETS-1, ↓CTGF,↑miR-499	OSF	Hou et al. (2015)
			Zheng et al. (2015)
			Wu et al. (2015)

Abbreviations: CS-Chondroitin Sulphate, DS-Dermatan Sulphate, HA-Hyaluronic Acid, KS-Keratan Sulphate, OSF- oral submucous fibrosis, UA-Uronic Acid.

The effect of capsaicin on the properties of collagen was studied using a combination of biophysical and computational tools. It was found that capsaicin strongly suppressed collagen fibril formation, increased the stability of collagen fibers in tendons, and had no effect on the stability of collagen. While capsaicin did not promote the disassembly of collagen fibrils it moderately protected the collagen fibrils from enzymatic degradation. However, computational studies have revealed the functions of the aromatic and amide regions of capsaicin in the capsaicin-collagen interaction. Capsaicin is bound to the “GFOGER”, “EKG”, and “GLO” triple-helical conformational sequences within Collagen-I to inhibit collagen fibril formation (Perumal et al., 2015) (Figure 2B).

## Capsaicin as an Anticarcinogenic Agent

The pro-carcinogenic effect of capsaicin in higher doses has been previously described (Murti et al., 1995) (Friedman et al., 2019). However, several studies have also demonstrated the potential anticancer effects of capsaicin (Ip et al., 2012; Gonzales et al., 2014; Clark and Lee, 2016; Cho et al., 2017; Dai et al., 2018; Friedman et al., 2018; Friedman et al., 2019; Mosqueda-Solis et al., 2021) and its non-genotoxic properties (Cho et al., 2017). Capsaicin was shown to reduce the incidence of oral epithelial dysplasia (OED) in a 4-nitroquinoline 1-oxide (4-NQO) induced tongue cancer model in male rats (Tanaka et al., 2002). Capsaicin prevents the transformation of OED to **o**ral **s**quamous **c**ell carcinoma (OSCC) in mice through the reduction of anti-apoptotic marker B-cell lymphoma 2 (BCL2) (Mohamed and AlQarni, 2019). In a recent systematic review by Mosqueda-Solís et al., on capsaicin intake and oral carcinogenesis, a definitive protective effect of capsaicin on the development of oral cancer in mice was demonstrated (Mosqueda-Solis et al., 2021). Additional evidence of capsaicin being an anticarcinogenic agent comes from the fact that stomach cancer is less common in the Indian subcontinent where the consumption of spicy food is not unusual (Barad et al., 2014). This is particularly beneficial among cisplatin-resistant stomach cancer cells whereby cisplatin inducible Aurora-A kinase is degraded by capsaicin to induce apoptosis (Huh et al., 2011).

**FIGURE 3:**
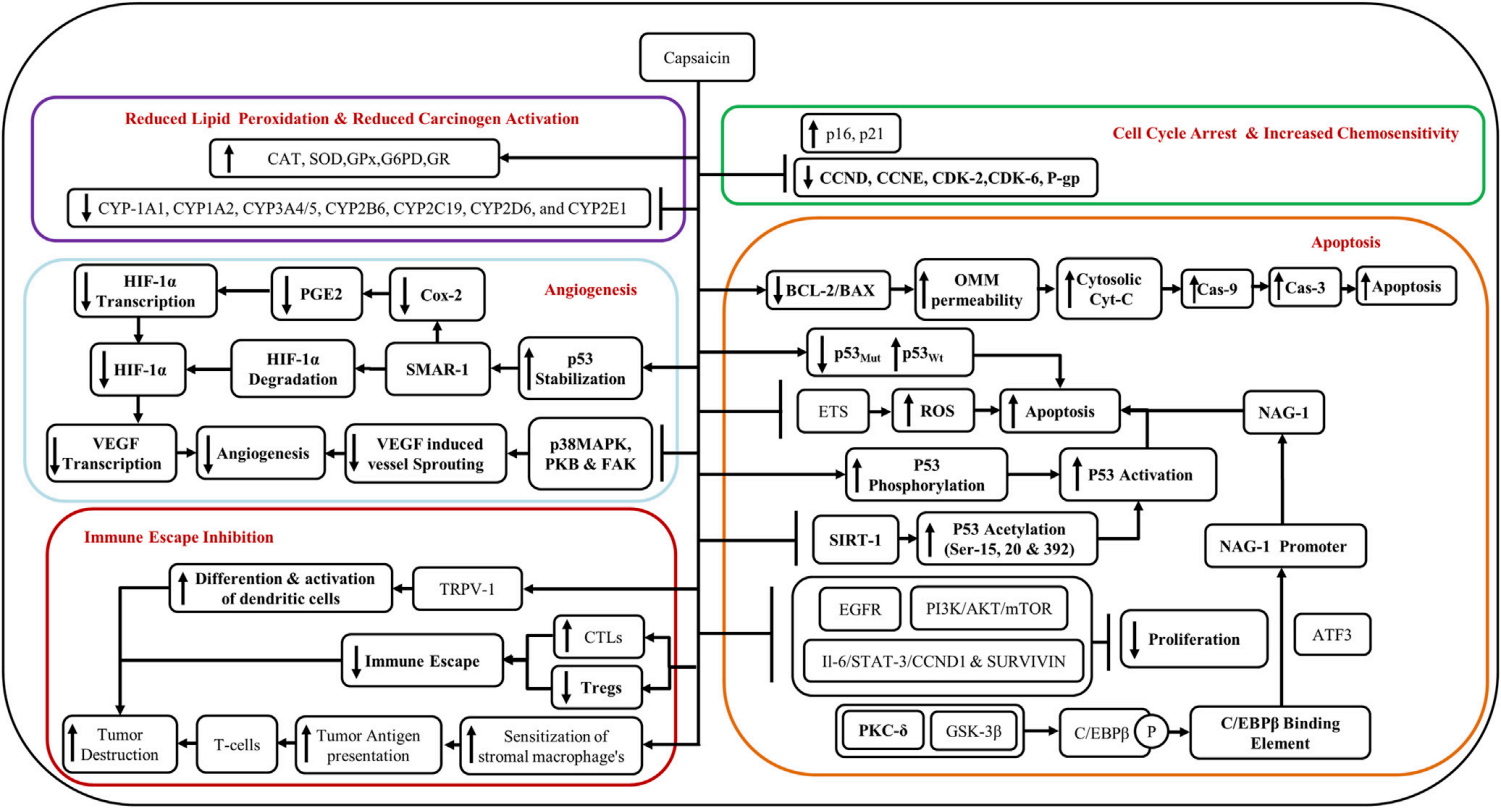
Schematic outline of the anti-malignant action of capsaicin through various pathways.

Capsaicin restores the wild-type p53 (p53_Wt_) and degrades mutant p53 (p53_Mut_) (Cho et al., 2017), and promotes apoptosis. Capsaicin decreases the BCL2/BAX ratio which in turn increases the permeability of the outer mitochondrial membrane (OMM) causing the release of cytochrome-c (Cyt-C) and its cytosolization. The cytosolic Cyt-C activates Caspase 9 (Cas-9) and Caspase 3 (Cas-3) which then cause apoptosis of the cell (Cho et al., 2017) (Figure 3).

Gonzales et al., in 2014 reported the anticancer effect of capsaicin on oral cancer cells through enhanced apoptosis, independent of the TRPV-1 receptor (Gonzales et al., 2014). Capsaicin has been shown to induce apoptosis in human tongue cancer cell lines SCC4, SCC25, and HSC3 via mitochondria-dependent and independent pathways (Ip et al., 2012; Gonzales et al., 2014). The apoptosis was mediated by p53 through enhanced phosphorylation at serine residues 15, 20, 392 and increased acetylation (through the inhibition of SIRT1) (Clark and Lee, 2016).

Another mechanism supporting the anticancer activity of capsaicin is via glycogen synthase kinase-3β (GSK3β)- and protein kinase C-δ (PKC-δ)-dependent phosphorylation of CCAAT enhancer-binding proteinβ (C/EBPβ). Phosphorylation of C/EBPβ augments the binding affinity of C/EBPβ onto non-steroidal anti-inflammatory drug (NSAID)-activated gene-1 (NAG-1) promoter, which activates the transcription of NAG-1 genes. Activating transcription factor 3 (ATF3) enhances the recruitment of C/EBPβ to the NAG-1 promoter by acting either as a bridge protein or by helping in the formation of a supramolecular complex along with other transcription factors. The activation of NAG-1 genes then triggers apoptosis (Lee et al., 2010; Clark and Lee, 2016) (Figure 3).

Additionally, capsaicin-induced a G0/G1 phase arrest in oral cancer cells through the reduction of cyclin-D (CCND), cyclin-dependent kinase 2 (CDK2), and cyclin-dependent kinase 6 (CDK6), and an increase in the levels of p21 and p16 (Ip et al., 2012). Furthermore, the anti-cancer effects of capsaicin have been demonstrated by the inhibition of Epidermal Growth Factor Receptor (EGFR) and PI3K/Akt/mTOR signaling pathways (Dai et al., 2018), and through IL-6/Signal Transducer and Activator of Transcription- 3 (STAT-3)/Cyclin D1 (CCND1) and survivin pathways (Cho et al., 2017) (Figure 3).

Capsaicin inhibits angiogenesis both *in vitro* and *in vivo*. It has been shown to cause the degradation of HIF-1α through the scaffold/matrix-associated region-binding protein 1 (SMAR1) and by the stabilization of p53 (Clark and Lee, 2016; Cho et al., 2017). SMAR1 upregulation leads to downregulation of Cox-2 and PGE-2 and reduced transcription of HIF-1α (Cho et al., 2017). Reduction in HIF-1α leads to reduced transcription of vascular endothelial growth factor (VEGF) and suppression of angiogenesis (Clark and Lee, 2016; Cho et al., 2017). Capsaicin inhibits VEGF-induced vessel sprouting and angiogenesis by the suppression of p38 MAPK, protein kinase B (PKB), and FAK pathways (Clark and Lee, 2016) (Figure 3).

The expression of the growth modulator, TRPV-1, was investigated in the pre-malignant and malignant lesions of the tongue. TRPV-1 expression was dramatically increased in all grades of oral squamous cell carcinoma (OSCC). An increase in TRPV-1 was also noted in the OSCC cell lines (Marincsak et al., 2009). This was, however, not due to the pro-carcinogenic effects of capsaicin as Ca^++^ channels were triggered minimally (evidence for the activation of the receptor) upon engagement of the receptor (Gonzales et al., 2014). Additionally, TRPV1 channels were inactive in primary tongue cancer-derived cell lines SCC4, SCC25, and HSC3 (Gonzales et al., 2014). Capsaicin seemingly had an anti-cancer effect in already transformed oral cells through a mechanism independent of TRPV1 (Gonzales et al., 2014), by acting as an antagonist of coenzyme-Q (Co-Q), inhibiting the electron transport system (ETS), thereby generating ROS, and inducing apoptosis (Figure 3). On the contrary, the immortalized keratinocyte cell line OKF6-TERT2 did demonstrate cytotoxicity to capsaicin via TRPV1 channels (Gonzales et al., 2014). Therefore, capsaicin has anticancer effects in the background of OSF via a TRPV1-dependent mechanism.

Hou et al. showed that the T/C + C/C genotypes and C allelic type for miR-499a produced reduced amounts of miR-499a when compared to the TT genotype or T allelic type (Hou et al., 2015). miR-499a negatively regulated the proto-oncogene ETS-1, which in turn upregulated the connective tissue growth factor (CTGF), a well-known orchestrator of fibrosis (Hou et al., 2015). The T/C + C/C genotypes and C allelic type for miR-499a had a greater risk for BQ-induced OSF than the TT genotype or T allelic type (Hou et al., 2015). The higher risk of fibrosis in T/C + C/C genotypes and C allelic type was due to decreased expression of miR-499a, which in turn caused the activation of ETS-1 and the upregulation of CTGF. The reduction in miR-499 expression was correlated with advanced stage and larger tumor size (Hou et al., 2015). Incidentally, mir-499a levels were restored by capsaicin (Zheng et al., 2015), which in turn caused a significant reduction in ETS-1 and CTGF. Thus, the restoration of miR-499 expression through capsaicin may avert the malignancy arising in OSF through downregulation of the ETS-1/CTGF pathway and degradation of androgen receptor (Wu et al., 2015; Zheng et al., 2015) (Figure 2C).

A reduction in the dendritic cell (DC) population in pre-malignant and malignant oral lesions with higher grades (Pellicioli et al., 2017), may promote malignancy through immune evasion. Several studies have shown the downregulation of immune response among those who chew areca nut (Wang et al., 2011; Wang et al., 2012; Chang et al., 2014; Lee et al., 2014). Areca nut extract (ANE) inhibits the differentiation and function of DCs (Wang et al., 2012; Lee et al., 2014). Furthermore, augmentation of myeloid-derived suppressor cells (MDSC) via continued areca nut use fosters malignancy through auxiliary downregulation of the immune response (Dolcetti et al., 2010; Wang et al., 2011). Notably, several studies have shown that capsaicin can restore the function of the immune system and stimulate the DCs (Gilardini Montani et al., 2015; Lee et al., 2016). Intertumoral depletion of CD8+T-cells/cytotoxic T cells (CTLs) and upregulation of regulatory T cells (Tregs) are also responsible for the immune escape of tumors (Cho et al., 2017). Capsaicin could counter the immune escape of tumors by upregulating the CTLs and downregulating Tregs. Moreover, capsaicin could also lead to enhanced sensitization of stromal macrophages to tumors and increase tumor antigen presentation thereby enhancing the destruction of tumors by T cells (Cho et al., 2017) (Figure 3).

## Future Perspectives

The debate over whether capsaicin is pro-carcinogenic or anti-carcinogenic as well as if it exerts pro-fibrotic or anti-fibrotic effects continue. There is overwhelming evidence confirming that capsaicin, when used in its purest form and at an appropriate dose, is anti-fibrotic and anti-carcinogenic. Repeated application of capsaicin causes the depletion of substance-P (a tumor promoter), thereby reducing inflammation. It has been established that capsaicin activates PPARγ through TRPV-independent mechanisms by activating the liver X receptors (LXR), which inhibit the activation of the NF-κB pathway via LXR response elements (LXREs). Blocking NF-κB activation suppresses the production of inflammatory cytokines like IL-1β, IL-6, and TNFα (Cho et al., 2017).

**FIGURE 4:**
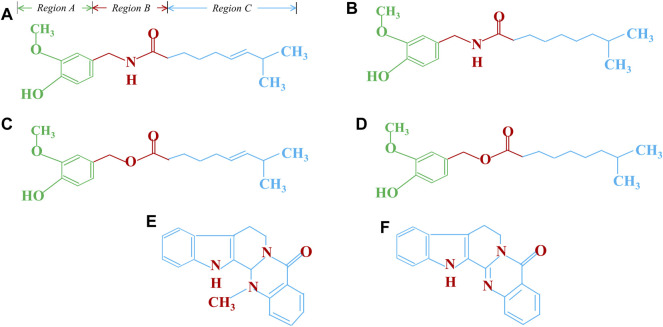
Chemical structure and activity relationship of capsaicin and its analogy **(A)**. Capsaicin, **(B)**. Dihydrocapsaicin, **(C)**. Capsiate, **(D)**. Dihydrocapsiate, **(E)**. Evodiamine, **(F)**. Rutaecarpine.

Capsaicin is anti-carcinogenic and thereby inhibits microsomal phase-I xenobiotic-metabolizing enzymes like CYP-1A1, CYP1A2, CYP3A4/5, CYP2B6, CYP2C19, CYP2D6, and CYP2E1, leading to reduced activation of carcinogens. Capsaicin inhibits the mutagenicity of tobacco-specific carcinogen 4-(methylnitrosamino)-1-(3-pyridyl)-1-butanone (NNK) by blocking its metabolic activation by microsomal enzymes (Cho et al., 2017). Reduced lipid peroxidation through the restoration of antioxidant enzymes like catalase (CAT), superoxide dismutase (SOD), glutathione peroxidase (GPx), glucose-6-phosphate dehydrogenase (G6PD), and glutathione reductase (GR) represent some other novel anti-cancer mechanisms mediated by capsaicin (Cho et al., 2017). Additionally, capsaicin and evodiamine restore chemosensitivity by inhibiting the P-Glycoprotein (P-gp) and ATP-binding cassette transporter G2 (ABCG2), which is an important drug efflux pump mediating chemoresistance (Cho et al., 2017; Friedman et al., 2018). The treatment of HSC-3 and SAS oral cancer cell lines expressing a mutant p53 gene with capsaicin, augmented chemosensitivity of chemotherapeutic agents like 5-FU, cisplatin, docetaxel and doxorubicin through increased autophagy and deceased ribophorin-II. This resulted in increased necroptosis of these cells as evidenced by elevated levels of necroptotic markers, the phospho-Mixed Lineage Kinase domain-like (MLKL) and phospho-Receptor interacting protein kinase 3 (RIP3) (Huang et al., 2021). The anticancer potential of capsaicin and its analogs is discussed in detail in Table 2 and their chemical structure have been shown in Figure 4. Structurally capsaicin has been divided into three regions aromatic, amide and hydrophobic regions which are designated region A, region B and region C, respectively. Capsaicin has been shown to act as a classic agonist of the TRPV-1 channel, while evodiamine is a potent selective agonist and differs from capsaicin in region C. Capsiate and Dihydrocapsiate differ from capsaicin in region B. The occurrence of a carbon-carbon double bond in region C in capsaicin and the lack of it dihydrocapsaicin, chemically differentiate from each other (Friedman et al., 2018) (Figure 4).

**TABLE 2:** Potential Anticancer action of capsaicin and its analogs.

Agent	Effect	Pathway/Mediators	References
I. Capsaicin	
↑ Antioxidant defense	↑ PI3K/Nrf2/HO-1 pathway	Clark and Lee (2016), Cho et al. (2017), Friedman et al. (2018)
↑ Antioxidant defense	↑GST, ↑QR and ↑HO-1	
↓ Lipid peroxidation	↑SOD, ↑CAT, ↑GPx, ↑GR, ↑ G6PD	
↑ Chemotherapeutic efficacy	↓P-gp	
↓ Inflammation	↓Substance-P, ↓IL-1β, ↓IL-6, ↓TNFα, ↓Cox-2, ↓NF-κB	
↓Activation of carcinogens	↓CYP1A1, ↓CYP1A2, ↓CYP3A4/5, ↓CYP2B6, ↓CYP2C19, ↓CYP2D6 and ↓CYP2E1	
II. Natural Capsaicin Analogues			
**Capsiate & Dihydrocapsiate**	↑ Apoptosis	↑ROS, ↑NF-κB ↓ ΔΨm	Friedman et al. (2018)
	↓ Angiogenesis	↓ Src Kinase, ↓ p125, ↓ p125, ↓ VE-Cadherin	
**Evodiamine and**			
**Rutaecarpine**	↑Apoptosis	↑p53 and ↑p21 cells	
	↑Autophagy	↑ ROS, ↑NO, ↑Bax,↑Ca^2+^, ↓ Bcl-2, ↓JNK	
	↓Angiogenesis	↓ MAPK and ↓ERK	
	↓Metastasis	↓MMP2,3, ↓JAK/STAT, ↓PGI	
**Resiniferatoxin**	↑Cell Cycle Arrest	↓ΔΨm, ↑PKC, ↑p21, ↓CCND1	
	↑Apoptosis	↓CoQ, ↓Mitochondrial Respiration	
**DihydroCapsaicin**	↑Apoptosis	↓ΔΨm, ↑Cyt-c, ↑Cytosolic Ca2+, ↑Cas-3 and 9	
	↑Autophagy	↑Catalase	
II. Capsaicin synergistic action with other compounds			
Combination	**Mechanism**	**Type of cancer**	Clark and Lee (2016)
**Capsaicin + Resveratrol**	↑ Apoptosis - p53-dependent NO elevation and enhanced caspase-3 activity	Colon Cancer	
**Capsaicin + Pirarubicin**	TRPV1 activation and inhibition of nuclear localization of PCNA	Bladder Cancer	
**Capsaicin + Genistein**	↑AMPK, ↓Cox-2	Breast Cancer	
**Capsaicin +3,3′-diindolylmethane**	Modulating the transcriptional activity of NF-κB, ↑OR↓ target genes downstream of NF-κB and p53	Colorectal Cancer	
**Capsaicin + Brassinin**	↓ MMP-2 and -9 expression and enzymatic activities	Prostate Cancer	
**Capsaicin+**			
**5-FU/cisplatin/docetaxel, doxorubicin**	↑phospho- MLKL, phospho- RIP3 and		
↑ necroptosis	HSC-3 and SAS oral cancer cell lines	Huang et al. (2021)	

Abbreviations: ΔΨm- Mitochondrial membrane potential, Cytochrome-c–Cyt-c.

## Conclusion

The role of chilies or spicy food in the genesis of OSF has long been debated. Capsaicin has a protective role in attenuating fibrosis and is potentially therapeutic in reversing conditions linked to collagen accumulation. The continuous burning pain observed in the mucosa of OSF patients is due to the activation of mast cells and P2X receptors. This pain further gets aggravated upon the activation of TRPV-1 channels by capsaicin. Higher doses of capsaicin, however, would result in the deactivation of TRPV-1 channels. An appropriate dosage of capsaicin may ameliorate fibrosis and prevent the malignant progression of OSF. Judicious use of capsaicin and its delivery as a topical agent may have therapeutic benefits in reducing the sensation of pain through receptor desensitization, by optimizing anti-fibrotic function, thus preventing the malignant transformation of OSF.

## Glossary

4-NQO: 4-nitroquinoline 1-oxide
ABCG2: ATP-binding cassette transporter G2
AMPK: AMP-activated protein kinase
AN: areca nut
ANE: Arecanut extract
ATF3: Activating transcription factor 3
BAX: BCL2 Associated X, Apoptosis Regulator
BCL2: B-cell lymphoma 2,
C/EBPβ: CCAAT enhancer-binding proteinβ
CaMKK-β: Ca2+/calmodulin-dependent protein kinase kinase-β
Cas-3: Caspase three
Cas-9: Caspase 9,
CAT: Catalase
CCND: Cyclin-D
CCND1: Cyclin D1
CCNE: Cyclin-E
CDK2: Cyclin-dependent kinase
CDK6: Cyclin-dependent kinase
Col-I: Collagen I
Co-Q: Coenzyme-Q
CTLs: Cytotoxic T cells
Cxs: Connexin
Cyt-C: Cytochrome-c
DC: Dendritic cell
EDV: Endothelium-dependent vasodilation
eNOS: Endothelial nitric oxide synthase
ERK2: Extracellular signal-regulated kinase
ETS: Electron transport system
Fn: Fibronectin
G6PD: Glucose-6-phosphate dehydrogenase
GPx: Glutathione peroxidase
GR: Glutathione reductase
GSK3β: Glycogen synthase kinase-3β
HDAC-5: Histone deacetylase-5
KLF2: Krüppel-like Factor 2
LXR: Liver X receptors,
LXRE: LXT response elements
MCD: Mast cell density
MCP-1: Monocyte Chemoattractant Protein-1
MDSC: Myeloid-derived suppressor cells
MEF-2: Myocyte enhancer factor-2
NAG-1: Non-steroidal anti-inflammatory drug (NSAID)-activated gene-1
NF-κB: Nuclear factor-kappa B
NGF: Nerve growth factor
NNK: 4-(methylnitrosamino)-1-(3-pyridyl)-1-butanone
NRF2: Nuclear factor erythroid 2-related factor 2
OMM: Outer mitochondrial membrane
OSF: Oral submucous fibrosis
p53Mut: Mutant p53
p53Wt: Wild-type p53
Panx: Pannexin
P-gp: P-Glycoprotein
PKB: Protein kinase B
PKC-δ: Protein kinase C-δ
PPAR-γ: Proliferator-activated receptor γ
PPAR-δ: Proliferator-activated receptor δ
SIRT-1: Sirtuin-1
SMAR1: Scaffold/matrix-associated region-binding protein 1
SOD: Superoxide dismutase
STAT-3: Signal Transducer and Activator of Transcription- three
Tregs: Regulatory T cells
TRPV-1: Transient receptor potential vanilloid
UCP2: Uncoupling protein 2
VEGF: Vasculoendothelial growth factor
